# Impact of Changing Drug Treatment and Malaria Endemicity on the Heritability of Malaria Phenotypes in a Longitudinal Family-Based Cohort Study

**DOI:** 10.1371/journal.pone.0026364

**Published:** 2011-11-03

**Authors:** Cheikh Loucoubar, Bronner Goncalves, Adama Tall, Cheikh Sokhna, Jean-François Trape, Fatoumata Diène Sarr, Joseph Faye, Abdoulaye Badiane, Alioune Badara Ly, Aliou Diop, Avner Bar-Hen, Jean-François Bureau, Anavaj Sakuntabhai, Richard Paul

**Affiliations:** 1 Institut Pasteur, Unité de Génétique Fonctionnelle des Maladies Infectieuses, Paris, France; 2 Laboratoire de Mathématiques Appliquées Paris 5 (UMR 8145), Université Paris Descartes, Paris, France; 3 Unité d'Épidémiologie des Maladies Infectieuses (UR 172, ED-SEV, Université Cheikh Anta Diop), Institut Pasteur de Dakar, Dakar, Senegal; 4 Ecole des Hautes Etudes en Santé Publique, Rennes, France; 5 Institut de Recherche pour le Développement, Laboratoire de Paludologie, Dakar, Senegal; 6 Laboratoire d'Études et de Recherche en Statististique et Développement, Université Gaston Berger, Saint-Louis, Senegal; 7 Center of Excellence for Vectors and Vector-Borne Diseases, Faculty of Science, Mahidol University, Bangkok, Thailand; Pennsylvania State University College of Medicine, United States of America

## Abstract

Despite considerable success of genome wide association (GWA) studies in identifying causal variants for many human diseases, their success in unraveling the genetic basis to complex diseases has been more mitigated. Pathogen population structure may impact upon the infectious phenotype, especially with the intense short-term selective pressure that drug treatment exerts on pathogens. Rigorous analysis that accounts for repeated measures and disentangles the influence of genetic and environmental factors must be performed. Attempts should be made to consider whether pathogen diversity will impact upon host genetic responses to infection.

We analyzed the heritability of two *Plasmodium falciparum* phenotypes, the number of clinical malaria episodes (*PFA*) and the proportion of these episodes positive for gametocytes (*Pfgam*), in a family-based cohort followed for 19 years, during which time there were four successive drug treatment regimes, with documented appearance of drug resistance. Repeated measures and variance components analyses were performed with fixed environmental, additive genetic, intra-individual and maternal effects for each drug period. Whilst there was a significant additive genetic effect underlying *PFA* during the first drug period of study, this was lost in subsequent periods. There was no additive genetic effect for *Pfgam*. The intra-individual effect increased significantly in the chloroquine period.

The loss of an additive genetic effect following novel drug treatment may result in significant loss of power to detect genes in a GWA study. Prior genetic analysis must be a pre-requisite for more detailed GWA studies. The temporal changes in the individual genetic and the intra-individual estimates are consistent with those expected if there were specific host-parasite interactions. The complex basis to the human response to malaria parasite infection likely includes dominance/epistatic genetic effects encompassed within the intra-individual variance component. Evaluating their role in influencing the outcome of infection through host genotype by parasite genotype interactions warrants research effort.

## Introduction

The genomics era has heralded a plethora of Genome Wide Association (GWA) studies that have successfully identified genetic determinants of many medical disorders [1]–[4]. Heritability analyses provide an indication of the genetic contribution underlying a specified phenotype. Whereas in the case of monogenic diseases genetic determinants in GWA studies account for the estimated heritability, there is considerable missing heritability in more complex diseases [5]. This had led to an intense debate of the potential causes for this, citing amongst others the potentially important roles of epistasis, gene-environmental interaction and the confounding effect of population specific genetic architecture [6]. In addition to genetic explanations, one potential source contributing to the missing heritability concerns the phenotype; poorly resolved phenotypes lower the power to detect genetic variants [7].

The application of GWA studies to infectious diseases has only more recently developed [8]–[10], but is likely to become increasingly implemented [11]. Infectious disease phenotypes are, however, composite phenotypes reflecting both the human and pathogen genetics and their interactions. Thus, the phenotype “problem” is likely to be much greater than in non-infectious diseases. Over the long-term, host-pathogen co-evolution will maintain genetic variation if the additive genetic value of a host genotype changes when parasites evolve in response to the selection induced by the host [12]. This, thus, may be apparent in the local genetic architecture of the human genetics determining specific traits, where populations have undergone widely different exposure to the pathogen. In addition, despite the current efforts to untangle the genetic basis to complex diseases [13], no attention has been paid to the impact of radical short-term changes in the pathogen population genetic structure, such as those induced by drug pressure, on the human genetic contribution to infection phenotypes.

In recent years, particular attention has been paid to addressing the human genetic susceptibility and resistance to *Plasmodium falciparum* malaria [14]–[16]. Sickle cell trait has long been recognized as having a protective effect against severe disease [17], [18] and this provided a positive control for the first GWA study of severe malaria [19]. Following this success and in the knowledge that the human genetic response to malaria parasite infection is complex and polygenic [20], it is now widely admitted that well-conducted epidemiological studies that take into account confounding environment factors are required [21]. In general, the requisite large sample size for GWA studies necessarily means combining participants from many sites. Whilst among-site variation in human population sub-structure and in the intensity of transmission can in principle be taken into account, such confounding variation may have more subtle effects. Variation in the intensity of transmission, for example, not only has discernable effects on the development of immunity, it also influences parasite genetic diversity [22].

To date genetic analyses have implicitly assumed that any variation brought about by parasite diversity will only have a minor impact, especially with very broad binary phenotypes such as severe versus mild malaria. This has been to some extent confirmed in animal models, but significant host-by-parasite interactions have been observed [23]. In contrast to such extreme binary disease phenotypes, there has been increasing interest in quantitative phenotypes that describe the outcome of infection [16], [24]–[27]. Such phenotypes focus on the actual biology of the parasite within the human host, rather than the extreme disease phenotype, but may be more affected by changes in parasite diversity. Parasite genetic variation in growth rate, transmissibility and other biological phenotypes is well recognized [28] and thus quantitative malaria phenotypes may be influenced strongly by parasite genetics. Indeed, it was recently demonstrated that there was a parasite genetic contribution to time to clearance following treatment [29]. Transmission intensity influences the number of different parasite clones within an infection, which itself can impact on quantitative phenotypes [30]. Moreover, malaria parasites exhibit extensive phenotypic plasticity and quantifiable parasite phenotypes are affected by the immunological and hematological state of the host [31]. Finally, parasite populations evolve over time, especially in the face of persistent drug pressure and there has been recent suggestion that drug resistance is linked to or will select for virulence of the parasite [32], [33]. All such sources of variation in the parasite population may significantly alter the observed outcome of infection and thus cloud the signal in the genetic analyses.

Here we address the extent to which malaria phenotypes in a longitudinal family-based epidemiological study are influenced by the changes in anti-malarial drug treatment and in transmission intensity from 1990 to 2008. We estimate the heritability of two *P. falciparum*-related phenotypes: the number of clinical malaria episodes (*PFA*) [16] and the proportion of infections carrying gametocytes (parasite stages that can infect mosquitoes) (*Pfgam*) [27], [34]. Heritability is an important parameter that determines statistical power in gene-mapping studies that use pedigree information. A large heritability implies a strong correlation between phenotype and genotype, so that loci with an effect on the phenotype can be more easily detected [35]. These two phenotypes were chosen to be representative of different types of phenotype: *PFA* will be strongly influenced by variation in transmission intensity, whereas *Pfgam* will more strongly reflect the host-parasite interaction. In addition to calculating the heritability, we estimate the shared environment (here house) and intra-individual (also known as “permanent environment”) effects, including maternal effects.

## Materials and Methods

### Ethics statement

The project protocol and objectives were carefully explained to the assembled village population and informed consent was individually obtained from all subjects either by signature or by thumbprint on a voluntary consent form written in both French and in Wolof, the local language. Consent was obtained in the presence of the school director, an independent witness. For very young children, parents or designated tutors signed on their behalf. The protocol was approved by the Ethical Committee of the Institut Pasteur de Dakar and the Ministère de la Santé et de la Prévention of Senegal. An agreement between Institut Pasteur de Dakar, Institut de Recherche pour le Développement and the Ministère de la Santé et de la Prévention of Senegal defines all research activities in the study cohorts. Each year, the project was re-examined by the Conseil de Perfectionnement of the Institut Pasteur de Dakar and the assembled village population; informed consent was individually renewed from all subjects.

### Study site and study population

The study was conducted in the malaria research project carried out since 1990 in a family-based cohort in Senegal, which has perennial holoendemic transmission (high force of infection). This site is managed by a tripartite agreement between the Institut Pasteur de Dakar, the Institut de Recherche pour le Développement and the Ministère de la Santé et de la Prévention of Senegal. A field research station with a dispensary run by nurses was constructed for the program and the health care is free-of-charge for the volunteers. All participants were asked to come to a study clinic for all their healthcare needs. Every person satisfying adhesion conditions could become a volunteer and every volunteer could leave the study at any time, therefore forming a dynamic open cohort. Further details of the study sites and adhesion criteria are previously described [36], [37].

The family structure (pedigree) was available after a demographic census performed for every volunteer at his adhesion in the project. A verbal interview of mothers or key representatives of the household was used to obtain information on genetic relationships between studied individuals, their children, their parents, and to identify genetic links among the population. The total pedigree comprised 828 individuals, including absent or dead relatives, composed of 206 nuclear families (father – mother couples with at least one child) with an average of 3.6 children each. In addition, previous typing with microsatellites has enabled the construction of a pedigree based on Identity-by-Descent (IBD) using MERLIN [16], [38].

### Data collection - P. falciparum malaria phenotypes

The parasite phenotypes analyzed were: (i) the number of *P. falciparum* clinical episodes per trimester (*PFA*) and (ii) the proportion of clinical episodes that were positive for gametocytes, parasite stages transmissible to mosquitoes (*Pfgam*). A malaria episode is defined as a clinical presentation with measured fever (axillary temperature >37.5°C) or fever-related symptoms (headache, vomiting, subjective sensation of fever) and with a blood smear positive for *P. falciparum* at a parasite/leukocyte ratio higher than the age-dependent pyrogenic threshold previously defined by Rogier et al. [39]. For *PFA*, we first excluded any observations of each trimester for which the individual concerned was not present for at least 30 days ( = 1/3 of the trimester). Individuals satisfying presence conditions without any *P. falciparum* clinical episode in a trimester were classified as *PFA = 0*; individuals satisfying presence conditions with 1 or more malaria clinical episodes in a trimester correspond to person-trimester with *PFA* = {1, 2, 3, 4, or 5}. Repeated clinical presentations within 15 consecutive days were not considered to be independent and were excluded from the analyses, unless there was a parasite negative blood smear between two clinical episodes. In all cases parasite positivity was established as follows. Thick and thin blood films were prepared and stained by 3% Giemsa stain. Blood films were examined under an oil immersion objective at ×1000 magnification by the trained laboratory technicians and 200 thick film fields were examined to count the number of asexual and gametocyte parasite stages. The proportion of clinical episodes carrying gametocytes excluded any repeated clinical presentations within 15 days of previous treatment.

The following covariates were considered: sex, house, season (4 categories: Jul–Sep; Oct–Dec; Jan–Mar; Apr–Jun) nested within year, year (5 categories: 1990 to 1994 for quinine period, 5 categories: 1995 to 1999 for 1^st^ chloroquine period, 4 categories: 2000 to 2003 to the 2^nd^ chloroquine period, 3 categories: 2004 to 2006 for fansidar period, 3 categories: 2006 to 2008 for ACT period) and logarithm of number of days present in each trimester. For *Pfgam*, we additionally considered the presence of other *Plasmodium* spp. parasites (*Plasmodium ovale* and *Plasmodium malariae*; 2 categories: yes/no) and time since last treatment. For *Pfgam*, age was found to be best described as a continuous variable in each drug period. By contrast, age classes <5 years, [5–15[, [15–35[ and ≥35 years best described the effect of age on *PFA*. Only individuals for whom there was pedigree information were included in the analysis.

### Data analyses

From 1990 to 2008, four different drug regimens were implemented: *Quinine* from 1990 to 1994, *Chloroquine* from 1995 to 2003, *Fansidar* from 2004 to mid-2006 and *Artemisinin-based combination therapy (ACT)* from mid-2006 to 2008. The chloroquine drug period was divided into before (CQ1) and after (CQ2) 1999. This was done both to reduce the chloroquine period data set size and to examine the chloroquine periods prior to and during the observed emergence of parasite resistance to this drug [40]. The statistical analyses were performed independently for each of the five drug treatment periods.

We implemented Generalized Linear Mixed Models (GLMM) using SAS 9.1.3 (SAS Institute Inc., Cary, NC, USA) procedures GLIMMIX, MIXED and INBREED [41]–[43]. GLMM allows fitting of mixed models with correlated random effects, such as those due to genetic relationships. Random effects are assumed to be normally distributed, and conditional on these random effects, the exogenous variable had (i) a Poisson distribution when the studied phenotype was number of *P. falciparum* clinical episodes per trimester (*PFA*) or (ii) a Binomial distribution when the studied phenotype was the proportion of clinical episodes that were positive for gametocytes (*Pfgam*). Genetic covariance, or relationship among all pairs of individuals in the study and among their parents or more distant ancestors, were stored in a squared matrix, the Pedigree-based genetic relatedness matrix, of dimension *K*×*K* where *K* is the total number of individuals in the pedigree including those with missing phenotypes. Genetic covariance between two individuals was computed using the pedigree information as described below:

For A and B, a given pair in a pedigree, the genetic covariance is computed as *r*(A,B) = 2× *coancestry*(A,B) where the *coancestry* between A and B is calculated using the method presented in Falconer and Mackay (1996) [44]: *coancestry*(A,B) = Σ*_p_*(1/2)*^n(p)^*×(1 + I *_Common Ancestor_*) where *p* is the number of paths in the pedigree linking A and B, *n(p)* the number of individuals (including A and B) for each path *p* and I_X_ is the inbreeding coefficient of an individual X, which is equal to the *coancestry* between the two parents of X. I_X_ is set to 0 if X is a founder. This matrix was built using INBREED procedure of SAS and then integrated into the models [42].

The objective of the model used for the analysis was to estimate and separate different sources of variation underlying the total variation observed for the phenotype: the relative contributions of human genetics (additive genetic variance), intra-individual variance, maternal effects, house effects and unexplained residual variation. The repeated measurements design allows us to separate additive genetic variance from intra-individual variance. The occurrence of related individuals living in different houses allows separation of additive genetic variance from that due to shared household. Therefore, the random part of the mixed models included (i) the house identification variable as random effect assuming independence between houses to capture variance due to houses, (ii) the individual identification variable twice: a first time to capture the additive genetic variance by assuming non-independence between individuals and using the subpart of the Pedigree-based genetic relatedness matrix concerning individuals for which the phenotype was observed as covariance matrix between all pairs and a second time to capture other individual variances (e.g. intra-individual effects) assuming independence between individuals and (iii) the mother identification variable to capture maternal effects, assuming non-independence between mothers and offspring, using the subpart of the Pedigree-based genetic relatedness matrix concerning mothers of individuals for which the phenotype was observed. The unexplained residual variation was then deduced.

*PFA* was analyzed using a Poisson regression model, which explicitly takes into account the non-negative integer-valued aspect of the dependent count variable. Therefore a GLMM with a Poisson distribution was fitted using SAS proc GLIMMIX and *log* as the link function between E(*PFA | covariates*) and a predictor that is linear. Initially a maximal model with all covariates was fitted and a minimal adequate model including only significant covariates was obtained. The effect of each covariate on the outcome variable was estimated taking into account both inbreeding, via the genetic relatedness matrix integrated in the SAS Proc GLIMMIX using the LDATA option, and repeated measures, as well as house effects.

The vector of random effects was assumed to follow a multivariate normal distribution:
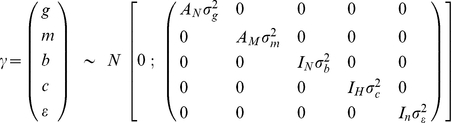
where *g* is the additive genetic effect, *m* is the maternal effect, *b* is the intra-individual effect, *c* is the house effect and *ε* is a random residual; *σ_g_^2^* , *σ_m_^2^* , *σ_b_^2^* , *σ_c_^2^* , *σ_ε_^2^* are the additive genetic, maternal, intra-individual, house and residual variances, respectively. A*_N_* represents the matrix of additive genetic relationships between individuals, with dimension *N*×*N*, A*_M_* represents the matrix of additive genetic relationships of mothers to offspring, with dimension *M*×*M*, I*_N_* is an identity matrix with dimension *N*×*N*, I*_H_* is an identity matrix with dimension *H*×*H*, and I*_n_* is an identity matrix with dimension *n*×*n*; and *n* = Σ*_i_n_i_* where *n_i_* is the number of measure for individual *i*, *N* is the number of individuals for which the phenotype was observed and *M* the number of their mothers.

The heritability is defined by *σ_g_^2^*/(*σ_g_^2^* + *σ_m_^2^* + *σ_b_^2^* + *σ_c_^2^* + *σ_ε_^2^*)

For each variance component, an estimate was also generated for each individual contributing to the overall component. Thus, for the additive genetic and intra-individual effects, an estimate was established for each person. Similarly for house and maternal effects, estimates were established for each house and mother.

*Pfgam* was analyzed by fitting a GLMM with a Binomial distribution, using SAS proc GLIMMIX [41]. The distribution of random effects and corresponding indices were defined as for *PFA* in the first analysis.

## Results

### Data description and epidemiological analyses of key environmental factors

**Figure 1:**
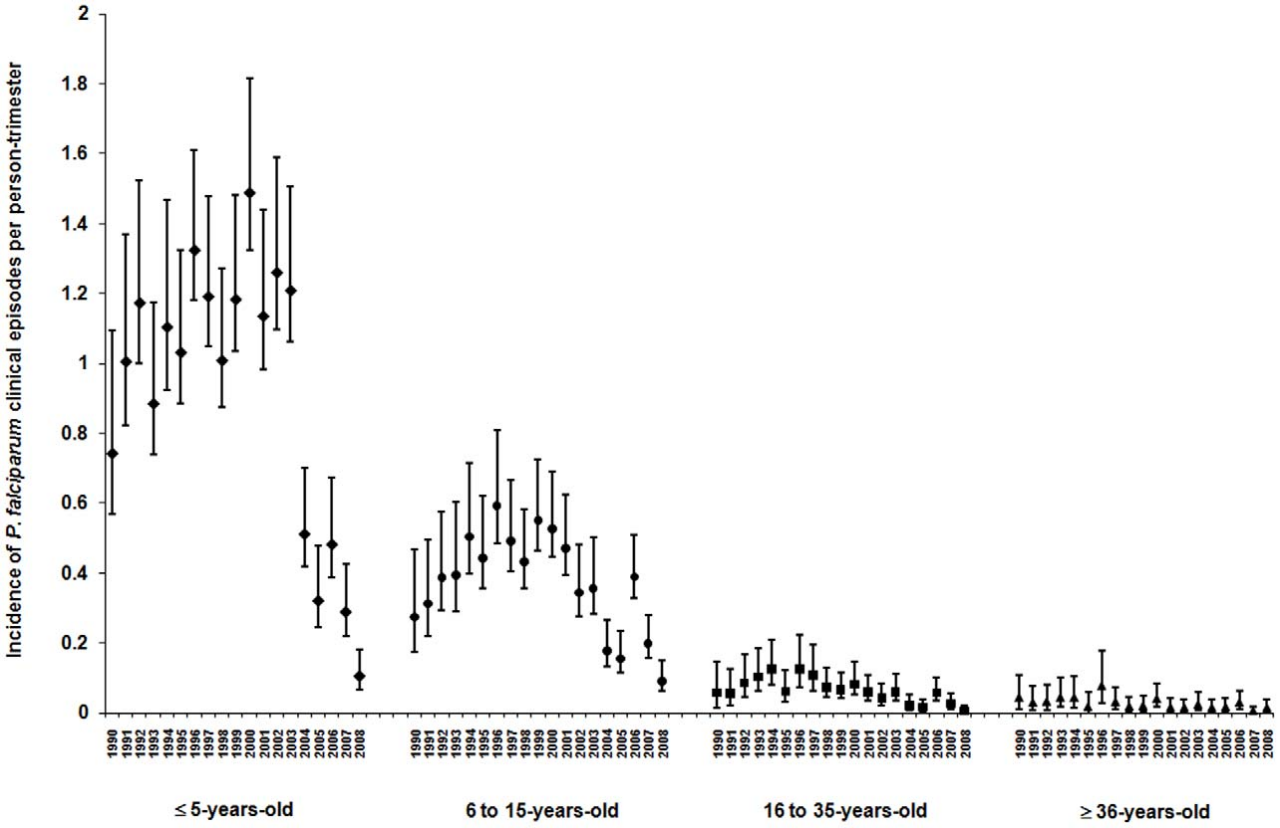
The incidence rate (mean and SEM) of clinical *P. falciparum* episodes per person-trimester (*PFA*) according to age classes (from left to right on the X-axis) <5 years, [5–15], [15–35] and ≥35 years that best describe the effect of age on *PFA*.

The first composite phenotype considered was the number of *P. falciparum* clinical episodes per person per trimester (*PFA*). Over the 19-year study period, 713 individuals were present from between one and 75 complete trimesters generating 22,169 person-trimesters of presence. There were a total of 5,680 clinical *P. falciparum* episodes. The maximum number of *PFA* per person-trimester was five and the median was one. 485 individuals had at least one *PFA* positive trimester during the study period. The maximum number of clinical episodes per person per drug period was 40 and the median was two. Table 1 summarizes the data by drug period and additionally gives the mean relatedness (by IBD) of the individuals present in each period. The number of clinical episodes decreased with age (P<0.0001) and this decrease was most accurately described by 4 groups (<5 years, 5–14 years, 15–34 years and >35 years old). Year and season also had a consistent influence on the number of clinical episodes (P<0.0001). The incidence rate of clinical episodes per trimester decreased significantly following the introduction of Fansidar; this change in the incidence rate is most evident in the most susceptible age group (<5 years of age) (Figure 1).

**Table 1:** Data summary for analyses of the number of *P. falciparum* clinical episodes per person per trimester (*PFA*) and the number carrying gametocytes (*Pfgam*).

Drug Period	Person-Trimesters	Individuals present	Mean relatedness	Number of Pf episodes	IndividualsPf positive	Range	% Pfgam positive	Individuals Pfgam positive	Range
Quinine	4080	338	0.0082	1454	234	1–40	37.2	117	1–16
CQ1	5469	405	0.0080	1950	245	1–38	47.1	151	1–26
CQ2	4800	423	0.0081	1481	205	1–38	48.6	155	1–28
Fansidar	3753	417	0.0084	466	148	1–11	17.1	55	1–5
ACT	4067	487	0.0083	329	135	1–10	12.2	34	1–3

Shown are the total number of person-trimesters per drug treatment period in which the number of *P. falciparum* clinical episodes occurred, the number of individuals present, their overall genetic relatedness (IBD), the number having a clinical episode, the range in the number of episodes per person, the percentage of these episodes that were positive for gametocytes, the number of individuals ever carrying gametocytes during a clinical episode and the range in the number of times individuals carried gametocytes.

The second composite phenotype considered was the number of *P. falciparum* clinical episodes that were positive for gametocytes, the parasite stage transmissible to mosquitoes. The prevalence of gametocytes at clinical presentation increased from 37% in the quinine period to 48% in both the chloroquine periods before decreasing to 17% and 12% in the Fansidar and ACT periods respectively (Table 1). The percentage of individuals ever gametocyte positive when having a clinical *P. falciparum* episode likewise increased from 50% in the quinine period to 75% in the second chloroquine period before decreasing to 37% and 25% in the Fansidar and ACT periods respectively. Age, as a continuous variable, was found to negatively associate with gametocyte presence during the quinine (P = 0.02), and the two chloroquine periods (P<0.001). Yearly variation had a significant impact in all periods except ACT. An increasing number of days of individual presence increased gametocyte carriage in the CQ1 period (P = 0.02) and increasing time since last drug treatment increased gametocyte carriage in the Fansidar period (P = 0.02).

### Heritability analyses – (i) number of P. falciparum clinical episodes per trimester

#### A. Additive genetic, intra-individual, maternal and house variance components

**Figure 2:**
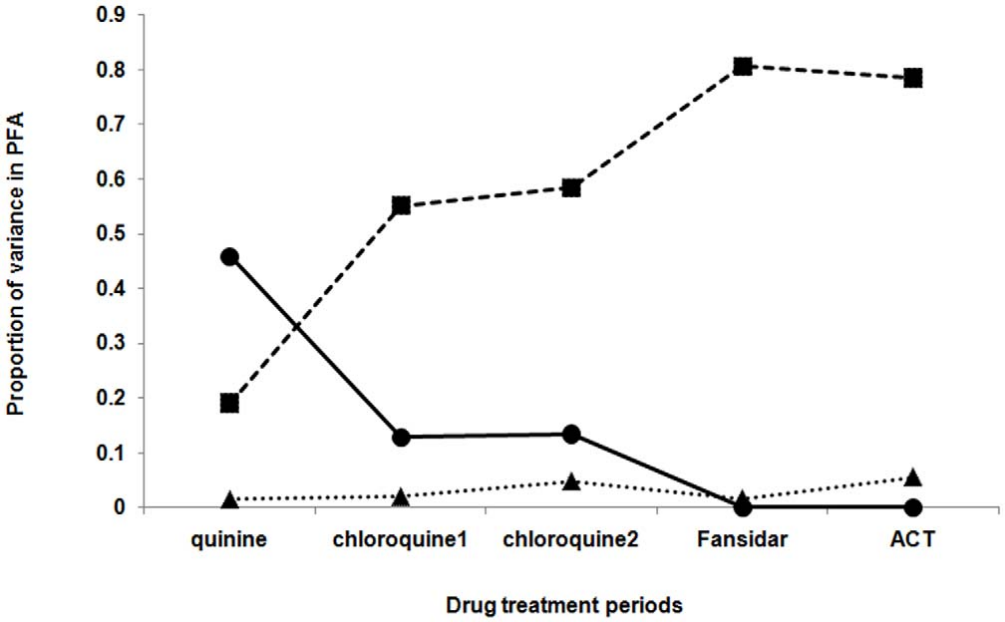
Proportion of variance in the number of clinical *P. falciparum* episodes per trimester explained by additive genetic (solid line), intra-individual (dotted line, squares) and house (thin dotted line, triangles) effects.

The narrow sense heritability of *PFA* was estimated by drug period. During the quinine period there was significant heritability, estimated at 46%, but which decreased and became non-significant in the subsequent drug treatment periods (Figure 2 and Table 2). Conversely, the intra-individual effect increased significantly following the quinine period, accounting for over 50% of the observed variance in *PFA*. There was no house effect during any period (Figure 2 and Table 2).

**Table 2:** Variance component analyses of the number of *P. falciparum* clinical episodes (*PFA*) according to drug period.

Drug period	var.comp	std.err	Z	Pr >Z	95% CI Inf	95% CI Sup
**Quinine**						
Genetic	0.941	0.384	2.450	**0.014**	0.189	1.693
Intra	0.391	0.247	1.580	0.057	0.152	2.343
House	0.030	0.106	0.280	0.390	0.003	8546
residual	0.692	0.016	43.410	<.0001	0.662	0.725
**Chloroquine 1**						
Genetic	0.257	0.205	1.250	0.211	−0.145	0.658
Intra	1.106	0.209	5.300	**<.0001**	0.789	1.664
House	0.039	0.059	0.670	0.252	0.007	85.995
residual	0.603	0.012	50.300	<.0001	0.580	0.627
**Chloroquine 2**						
Genetic	0.281	0.242	1.160	0.246	−0.193	0.756
Intra	1.230	0.229	5.370	**<.0001**	0.880	1.838
House	0.101	0.109	0.930	0.177	0.026	6.787
residual	0.493	0.011	46.870	<.0001	0.473	0.514
**Fansidar**						
Genetic	0.000	-	-	-	-	-
Intra	1.797	0.214	8.380	**<.0001**	1.441	2.304
House	0.036	0.059	0.610	0.272	0.006	392.83
residual	0.395	0.010	41.290	<.0001	0.377	0.415
**ACT**						
Genetic	0.000	-	-	-	-	-
Intra	1.759	0.208	8.450	**<.0001**	1.413	2.250
House	0.125	0.096	1.300	0.098	0.042	1.390
residual	0.357	0.008	43.240	<.0001	0.341	0.374

Genetic – additive genetic effect; Intra – Intra-individual effect; House – House effect.

The intra-individual effect includes, amongst other parameters, any maternal contribution, whether genetic or environmental. In the case of malaria parasite infection, for example, maternal antibodies protect the newborn during the first few months of life and thus the mother transfers her acquired immunity. In addition, infection during pregnancy can lead to low birth weight with consequent effects on health of the newborn and potentially later in life [45]. Thus, as classically performed in heritability analyses, we consequently evaluated the contribution of a maternal effect in addition to the additive genetic and intra-individual effects. There was no maternal effect during any drug period.

#### B. Additive genetic and intra-individual estimates for individuals

Estimates for the additive genetic variance strongly correlated for all the three drug periods for which the total additive genetic variance was not zero (i.e. thus for which there were non-zero genetic estimates per individual). There were only individual significant estimates for the additive genetic effect during the quinine period. Nineteen individuals had significant estimates during the quinine period; fourteen of these were present during more than one drug period but none had significant estimates subsequent to the quinine period. By contrast, five of them had significant estimates for the intra-individual effect in periods subsequent to the quinine period. Overall, individual estimates of genetic effects were highly correlated with intra-individual effects by drug period when non-zero (i.e. for quinine, CQ1 and CQ2 periods, Table 3) (r = 0.73, 0.71 and 0.65 respectively).

**Table 3:** Correlation of individual estimates of (i) the intra-individual and (ii) additive genetic effects underlying the variation in the number of *P. falciparum* clinical episodes according to drug period.

*PFA*					
(i) Intra	Quinine	CQ1	CQ2	Fansidar	ACT
Quinine		0.49***	0.04	−0.01	0.04
CQ1			0.30***	0.002	0.04
CQ2				0.29***	0.18*
Fansidar					0.16*

(ii) Genetic	Quinine	CQ1	CQ2		
Quinine		0.51***	0.23***		
CQ1			0.44***		

*P<0.05,

**P<0.01,

***P<0.001.

By definition, major components of the intra-individual variance are features that are particular to each individual. Pertinent to malaria parasite infection would be heterogeneity in exposure to mosquitoes but that which is independent of any detectable household spatial effect; i.e. specific individual behaviors that lead to differential exposure to mosquitoes. We examined how the intra-individual estimates for each individual were correlated over the drug periods. Estimates always correlated in the drug period that followed, but decreasingly so in subsequent drug periods (Table 3). Estimation of the individual contributions to the overall intra-individual effects revealed that 54, 47, 91 and 76 individuals had significant estimates in the CQ1, CQ2, Fansidar and ACT periods respectively. There were no individuals with significant estimates during the quinine period. The majority of these individuals (129 of 191) had a significant estimate in only one drug period. Fifteen and 47 individuals had significant estimates in three and two drug periods respectively.

**Figure 3:**
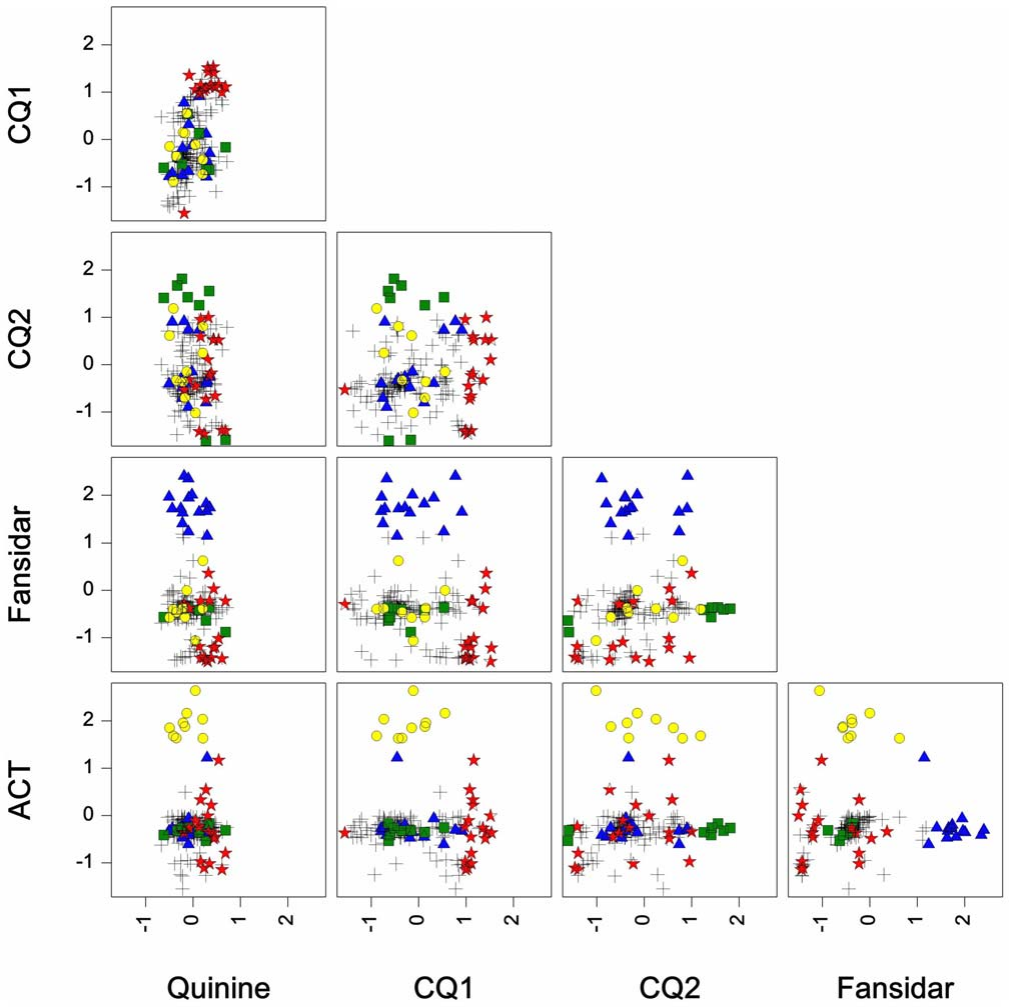
Comparative scatter plot of the Intra-individual estimates per individual per drug period for those individuals present throughout the study period. Individuals with significant intra-individual estimates at any period are shown in color: red stars (significant in CQ1), green squares (significant in CQ2), blue triangles (significant in Fansidar) and yellow circles (significant in ACT).

Of the 210 individuals present throughout the 19 year period, 69 had significant intra-individual estimates: fifty individuals in only one treatment period and the remainder in two (n = 15) or three (n = 4) different periods. Figure 3 displays a comparative scatter plot of intra-individual estimates in all drug periods. For simplicity, only the 50 individuals with significant estimates during a single drug period are highlighted: individuals with a significant estimate in a specific period are denoted as red stars (CQ1), green squares (CQ2), blue triangles (Fansidar) and yellow circles (ACT) in every graph. In the vertical quinine box column, all points cluster around zero with respect to the x-axis – there is no intra-individual effect in the quinine period. This negligible intra-individual variance component in the quinine period and the subsequent increase in the following periods can be clearly seen in Figure 3: the data points are increasingly spread out along the x-axis from the quinine column through the CQ1, CQ2 and Fansidar columns. The extreme significant values in the CQ1 (red stars), CQ2 (green squares), Fansidar (blue triangles) and ACT (yellow circles) periods clearly separate from the rest in their respective drug periods: thus for example the individuals represented by yellow circles have much larger values than the others in the ACT Y-axis row, whereas these same individuals do not differ from the rest in the CQ1, CQ2 and Fansidar Y-axis rows. This shows in detail how individuals with much higher or lower numbers of *P. falciparum* episodes (very positive or very negative values) have so in only single drug periods. Interestingly, the degree to which the significant points separate from the rest appears to increase with time (i.e. from CQ1 through ACT); the blue triangles (Fansidar) and yellow circles (ACT) are more clearly separated from the rest in their respective Y-axis rows. This increase in the intra-individual variance component as displayed though individual estimates over time is reflected in the summarized intra-individual variance component in Table 2. This shows that as the overall incidence rate drops, there is a growing gap between certain individuals having a high numbers of episodes and the rest. Comparing across drug periods, not only do period-specific significant individual estimates become non-significant in subsequent periods, they seemingly take on increasingly opposed values. This is most evident for CQ1, where the significant estimates for this period, denoted by red stars, decrease in value during the CQ2 and Fansidar periods (Figure 3, horizontal row “ACT”). Similarly for CQ2, significant estimates (green squares) became less than zero in the Fansidar and ACT periods. This suggests that individuals with previously very high numbers of clinical episodes have increasingly fewer numbers of episodes than the rest. One explanation for this would simply be the acquisition of clinical immunity due to repeated exposure to the parasite.

As can be seen in Figure 1, age is a reasonable proxy of the acquisition of immunity and both age and time spent within the site impact upon incidence rate. However, no single factor was found to be shared by individuals with significant intra-individual estimates. I.e. Age, gender and time spent within the village since inception of the study or during the six months prior to the episode were not significant variables determining the intra-individual estimate.

In the knowledge that resistance to chloroquine and then Fansidar emerged during the respective drug treatment periods, a potentially confounding factor would clearly be repetitive presentation of a single infection because of treatment failure. To evaluate whether the observed increases in the intra-individual variance was a result of drug treatment failure, we examined whether individuals with significant individual intra-individual estimates had a shorter time since previous treatment in the quinine and chloroquine periods, when incidence rate remained high and stable. Although the time since previous treatment for those individuals having significant intra-individual estimates at any time was shorter than for those never having significant estimates (P<0.001), drug period *per se* had no effect (P = 0.31). Thus, there was no difference in time between infections in the quinine and 2 chloroquine periods, suggesting that treatment failure was not causing this significant increase in the intra-individual variance component.

### Heritability analyses – (ii) prevalence of gametocytes during clinical P. falciparum episodes

#### A. Additive genetic, intra-individual, maternal and house variance components

**Figure 4:**
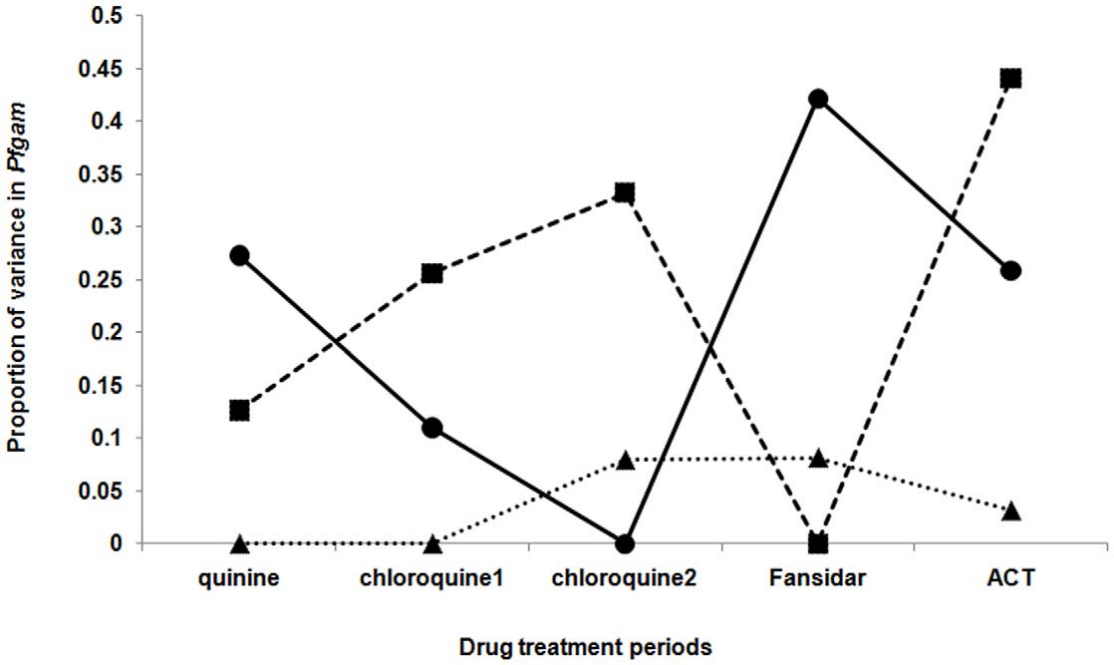
Proportion of variance in the prevalence of *P. falciparum* gametocytes during clinical *P. falciparum* episodes (*Pfgam*) explained by additive genetic (solid line), intra-individual (dotted line, squares) and house (thin dotted line, triangles) effects.

Heritability for the prevalence of gametocytes during clinical presentation only approached significance during the Fansidar period (P = 0.057) (Table 4, Figure 4). By contrast, the intra-individual effect increased significantly during the chloroquine periods, before becoming non-significant in the Fansidar and ACT periods. There were no house or maternal effects.

**Table 4:** Variance component analyses of the prevalence of gametocytes in treated clinical episodes (*Pfgam*) according to drug period.

Drug period	var.comp	std.err	Z	P-value	95% CI Inf	95% CI Sup
**Quinine**						
genetic	0.423	0.317	1.340	0.181	−0.197	1.044
Intra	0.196	0.272	0.720	0.236	0.040	156.760
House	0.000	.	.	.	.	.
residual	0.932	0.040	23.390	<.0001	0.858	1.015
**Chloroquine 1**						
genetic	0.164	0.195	0.840	0.401	−0.218	0.545
Intra	0.380	0.218	1.750	**0.041**	0.159	1.814
House	0.000	.	.	.	.	.
residual	0.942	0.035	27.300	<.0001	0.878	1.013
**Chloroquine 2**						
genetic	0.000	.	.	.	.	.
Intra	0.530	0.119	4.440	**<.0001**	0.356	0.870
House	0.127	0.090	1.410	0.079	0.045	1.050
residual	0.936	0.031	30.010	<.0001	0.878	1.001
**Fansidar**						
genetic	0.658	0.346	1.900	0.057	−0.021	1.336
Intra	0.000	.	.	.	.	.
House	0.127	0.219	0.580	0.281	0.021	3389.110
residual	0.773	0.055	14.150	<.0001	0.677	0.893
**ACT**						
genetic	0.570	1.224	0.470	0.641	−1.829	2.970
Intra	0.973	1.035	0.940	0.174	0.250	58.229
House	0.070	0.453	0.150	0.439	0.007	2.5E+65
residual	0.593	0.052	11.500	<.0001	0.503	0.708

Genetic – additive genetic effect; Intra – Intra-individual effect; House – House effect.

#### B. Additive genetic and intra-individual estimates for individuals

Correlation between estimates for the individual intra-individual and genetic effects revealed a similar pattern to *PFA*: there was significant correlation between estimates in consecutive drug periods, both with respect to estimates of individual intra-individual and additive genetic effects, but no correlation between more distantly related periods (Table 5). Moreover, individual estimates of the genetic and intra-individual effects by drug period were again highly correlated when non-zero (i.e. for Quinine, CQ1, and ACT periods, Table 5) (r = 0.79, 0.77 and 0.80 respectively).

**Table 5:** Correlation of individual estimates of (i) the intra-individual and (ii) additive genetic effects underlying the variation in the proportion of *P. falciparum* clinical episodes positive for gametocytes according to drug period.

*Pfgam*					
(i) Intra	Quinine	CQ1	CQ2	Fansidar	ACT
Quinine		0.23*	0.42***	-	0.33
CQ1			0.26**	-	0.11
CQ2				-	0.34**

(ii) Genetic	Quinine	CQ1	CQ2	Fansidar	ACT
Quinine		0.31**	-	0.40*	0.27
CQ1			-	0.33**	−0.02
CQ2				-	-
Fansidar					0.25*

*P<0.05,

**P<0.01,

***P<0.001.

The strongly significant intra-individual variance component in CQ2 was due to 12 individuals, eight of whom repeatedly had gametocytes and four who rarely presented with gametocytes. Although the time since previous drug treatment was shorter in these significant individuals, there was no difference between those frequently carrying gametocytes and those rarely doing so (mean 32.4 days SEM 2.5 *vs*. 34.8 days SEM 2.02). There is thus no indication that previous drug treatment is causing this intra-individual effect. No obvious factor, such as age or sickle cell trait, was shared by such individuals. Five of these individuals had significant intra-individual estimates for PFA. Only one individual had a significant intra-individual estimate in the CQ1 period and was not significant in the CQ2 period.

### Correlations between malaria phenotypes

There were no significant correlations in either individual additive genetic or intra-individual effects between *PFA* and *Pfgam* at any period where non-zero estimates were available.

## Discussion

Here we have made an initial study of the heritability of two *P. falciparum* malaria-related phenotypes in a single population over time. The analyses divided the longitudinal study according to drug treatment to examine the impact of the radical selection pressure that would have been exerted on the parasite population at each change in drug treatment. In addition, the change in transmission intensity occurring over the 19 year enabled us to assess its impact on the heritability of the malaria phenotypes. The evolution of anti-malarial drug resistance and the force of infection have been well studied in the population [36], [37], [40] and thus we explored heritability in a single population undergoing well-defined environmental changes.

Firstly, it was notable that for *PFA*, a phenotype known to be influenced by human genetics, significant heritability was lost following the change in drug treatment from quinine to chloroquine and in subsequent drug periods. There was no significant change in incidence rate, at least during the quinine and chloroquine periods, no difference in the number of different individuals presenting with clinical disease, or in the pedigree structure (as estimated by the mean genetic relatedness). This suggests that the implementation of the new drug in some way interfered with the human genetic contribution to the outcome of infection. In direct contrast, the intra-individual variance component increased following the implementation of chloroquine.

Intra-individual variance encompasses effects specific to each individual, classically including maternal effects and dominance (non-additive) genetic effects [35], [46]. There was no maternal effect for the number of *P. falciparum* clinical episodes in our cohort at any time period. The very high correlation of the individual genetic and intra-individual estimates within each drug period suggests that the two effects are highly confounded. This might be a result of insufficient resolution of the relatedness matrix within each drug period – i.e. either not enough relative-pairs were present within each period and/or the IBD matrix was not sufficiently resolved. This would lead to confounding between shared environmental, additive and non-additive genetic effects [47] and might explain the loss of heritability. However, given the similarity in mean genetic relatedness of individuals in the quinine (when the genetic effect was significant) and other periods, this seems an insufficient explanation. One potential source of variation would be local heterogeneity in individual exposure to mosquitoes. The increase in the intra-individual variance component as the transmission intensity decreased is consistent with heterogeneity in mosquito biting. Although there was no evidence for a significant impact of shared environment (house), heterogeneity in exposure may occur at a finer level of spatial resolution and/or that reflecting individual behavioral differences ([48] including commentary). One possible source of differential exposure would come from bednet use. However, long-lasting insecticidal-treated nets were not actively promoted until the summer of 2008. Individuals showing extreme intra-individual estimates shared no particular feature, whether it be age, sex or time present in the study site. This argues against any particular behavior or state of immunity contributing to the observed increase in estimates. The intra-individual variance component also includes environmental effects on an individual's phenotype that are constant across (or common to) repeated measures on that individual [46]. It is notable that not only do individual estimates correlate only with those from the subsequent drug period, but also that the majority of the extreme values per individual occurred in one drug period. One explanation for this concerns the impact of the differing drug treatments on the parasite population.

The most evident change in the parasite population during the study was the development of resistance first to chloroquine and then to Fansidar [40]. Treatment failure would result in the same individual presenting more than once for the same infection, thus artificially increasing that individual's number of malaria episodes and hence the estimated intra-individual effect. However, there was no evidence for treatment failure biasing the number of malaria episodes per person. The second effect of drug pressure would be to radically reduce parasite diversity and select for a sub-population of parasites. This process would not be instantaneous, because the majority of the parasite population at any one time in this cohort resides in untreated, asymptomatic infections. Thus, the positive correlations of individual intra-individual and indeed additive genetic estimates in consecutive drug periods might reflect the slowly changing parasite population, implicitly suggesting the existence of specific human-parasite interactions. Drug pressure would result in a stochastic loss of particular parasite genotypes, selection for drug resistant genotypes and potentially selection of parasites more pathogenic for particular individuals. The changing drug regimens would be expected to differentially select for parasite genotypes at each instance, thus making it highly unlikely that the same individuals would be continually susceptible. Whilst an attractive hypothesis, a combination of immune state, behavior and random focal transmission for specific periods of time could generate the observed increase in the intra-individual effect. Our study can not provide the immunological and parasite genetic data that demonstrate changes in the parasite population that would likely have clinical implications for a sub-set of individuals. Moreover, given the complexity and uncertainty of the key parasite antigens that are implicated in the development of clinical immunity [49], such data might not be simple to interpret.

In contrast to the immeasurable effect of very fine scale spatial heterogeneity in exposure to infection that will impact on *PFA*, variability in gametocyte production in an infection will reflect the influence of the host-parasite interaction. Both parasite and host genetics can influence gametocyte production [27], [50]. In this study we found no additive genetic effect underlying the proportion of clinical infections with gametocytes, confirming our previous observations [27]. Interestingly, however, there was a similar increase in the intra-individual effect to that observed for *PFA* and the two phenotypes were not correlated. Moreover, as for *PFA*, there was good correlation in estimates across only consecutive periods. These comparable effects to *PFA* were particularly notable during the period when transmission intensity was stable. Subsequently, the decrease in intensity in the Fansidar and ACT periods was accompanied by an even more significant decrease in gametocyte prevalence, resulting in perilously small sample sizes for reliable analysis.

Here, the period of drug treatment strongly influenced this phenotype. Such an influence has been well documented following treatment. Chloroquine increases gametocyte production [51] and Fansidar has also been suggested to increase gametocyte production [52] and/or longevity of gametocyte carriage in a single infection with drug resistant parasites [53]. By contrast, ACT has a gametocytocidal activity and reduces gametocyte carriage [54]. Here, there were no indications that previous treatment contributed to gametocyte presence at presentation, thereby inflating the intra-individual effects in the chloroquine periods. During the Fansidar period, a longer time since treatment was associated with gametocyte presence. The variation in the prevalence of gametocytes at presentation strongly suggests that the parasite population altered according to drug period and the correlated individual intra-individual estimates over successive drug periods are similar to those seen for *PFA*. This would support the hypothesis that changes in the parasite population diversity are contributing to the observed phenotype.

Estimation of heritability in its broad sense in natural populations is not possible and hence narrow sense heritability, which estimates the additive genetic contribution, is calculated. Actual values of heritability are specific for a study population at a particular time and thus strict comparison is not informative, although broad trends can be inferred. The size of heritability provides an indication of the power to detect the effect of individual genes when performing GWA studies. Here it is clear that for several reasons, the choice of the study period for GWA study analysis will affect the quality of the signal. The requirement for large longitudinal data sets to generate sufficient power must therefore be offset by the ever-increasing noise that accompanies long-term data sets – more time means more variance [55].

The peculiarity of the variance component analyses in this study was the replacement of an additive genetic component by an intra-individual component over time. Classical components of the intra-individual component, such as maternal effects, were not found to be the root cause of this and spatial heterogeneity in exposure seems an insufficient explanation, especially during the quinine and chloroquine periods. Insufficient resolution and power of the pedigree matrix may have led to confounding between additive and non-additive genetic components, but again this seems an inadequate explanation given the mean genetic relatedness of the individuals implicated. Observed patterns of individual estimates were consistent with there being specific host-parasite interactions. Although relatives might be expected to respond similarly to an identical parasite, this might not be detectable as an additive genetic component. To what extent changes in the parasite population can impact upon genetic studies is important to understand, both on a practical level of study sampling strategy and at a fundamental level to ask whether candidate genes should be expected to have an effect under whatever circumstances. In the hypothetical case of population fixation of a protective gene, heritability will be zero. What will be the expected heritability in a diverse human population if parasite diversity approaches zero? Will certain genes only be protective against a sub-set of parasites?

In this study we have found suggestive evidence that the parasite population may impact upon estimates of heritability. Whereas a review of theory and data have led to the suggestion that additive genetic variance will represent the majority of genetic variance in complex traits [56], this conclusion averages across populations and may not therefore be the case within a single population [6], especially in the case for infectious diseases. The complex, polygenic basis to the human response to malaria parasite infection may well include dominance/epistatic genetic effects that are encompassed within the intra-individual effect. Evaluating their role in host genotype by parasite genotype interactions in model systems will surely be fruitful. In conclusion, prior genetic analysis of carefully defined phenotypes, both spatially and temporally delimited, must surely not only be a pre-requisite to more detailed GWA studies, but also may be informative for the potential importance of pathogen genetics and the occurrence of host-pathogen interactions.
